# The Cell Type–Specific Functions of miR-21 in Cardiovascular Diseases

**DOI:** 10.3389/fgene.2020.563166

**Published:** 2020-11-20

**Authors:** Beibei Dai, Feng Wang, Xiang Nie, Hengzhi Du, Yanru Zhao, Zhongwei Yin, Huaping Li, Jiahui Fan, Zheng Wen, Dao Wen Wang, Chen Chen

**Affiliations:** ^1^Division of Cardiology, Department of Internal Medicine, Tongji Hospital, Tongji Medical College, Huazhong University of Science and Technology, Wuhan, China; ^2^Hubei Key Laboratory of Genetics and Molecular Mechanisms of Cardiological Disorders, Wuhan, China

**Keywords:** miR-21, cardiovascular diseases, cardiomyocytes, fibroblast, endothelial

## Abstract

Cardiovascular diseases are one of the prime reasons for disability and death worldwide. Diseases and conditions, such as hypoxia, pressure overload, infection, and hyperglycemia, might initiate cardiac remodeling and dysfunction by inducing hypertrophy or apoptosis in cardiomyocytes and by promoting proliferation in cardiac fibroblasts. In the vascular system, injuries decrease the endothelial nitric oxide levels and affect the phenotype of vascular smooth muscle cells. Understanding the underlying mechanisms will be helpful for the development of a precise therapeutic approach. Various microRNAs are involved in mediating multiple pathological and physiological processes in the heart. A cardiac enriched microRNA, miR-21, which is essential for cardiac homeostasis, has been demonstrated to act as a cell–cell messenger with diverse functions. This review describes the cell type–specific functions of miR-21 in different cardiovascular diseases and its prospects in clinical therapy.

## Introduction

According to the 2013 global burden of disease study (GBD), 17.3 million individuals died due to cardiovascular diseases (CVDs) worldwide. It causes twice as many deaths as cancer, contributing to 31.5% of total deaths and 45% of deaths due to non-infectious disease (Townsend et al., 2016). The unmanageable factors of CVDs comprise family history, sex, and age, and the manageable factors comprise cigarette smoking, dyslipidemia, diabetes mellitus, sedentary lifestyle, unhealthy diet, and stress. The most common types of CVDs are acute myocardial infarction (AMI), arrhythmias, vascular diseases, cardiomyopathy, and heart failure (HF) (Olson, 2014). With the development of certain effective drugs and devices, the incidence rates of CVDs were controlled to a certain extent. However, no significant improvements in overall outcomes have been observed, which necessitates further insights into molecular and pathological features of the diseased vessel and heart along with innovative therapeutic strategies (MacKenna et al., 2000; Bruneau, 2008). Lately, microRNAs (miRNAs), a kind of conserved small non-coding RNA (ncRNAs), have garnered interest as critical regulators of CVDs.

The biogenesis of miRNAs is a multistep process. Briefly, they are first transcribed by RNA polymerase II into primary-miRNAs (pri-miRNAs). The pri-miRNA hairpin is then excised in the nucleus by complexes that contain the RNase III enzyme Drosha and the RNA-binding protein DiGeorge syndrome critical region 8 (DGCR8). Drosha recognizes the junction at the base of the hairpin (the junction formed by double-stranded RNA–single-stranded RNA), and the two DGCR8 proteins bind to the stem and ensure proper cleavage. The pre-miRNA hairpins are composed of ∼70 nucleotides, whose ends are characterized by a 2′ nucleotide overhang of the 3′ end, a 5′ phosphate, and a 3′ hydroxyl at the 3′ end, which are recognized by Exportin 5 (XPO5) and transferred into the cytoplasm. In the cytoplasm, the RNase III enzyme Dicer binds to pre-miRNAs by identifying the structures comprising the 3′ overhang, 5′ phosphate, and the loop and cuts the pre-miRNAs at a length that is species-specific and produces a mature miRNA duplex with another classical 2-nucleotide overhang of the 3′ end.

One strand of the mature miRNA duplex (also called the guide strand) binds to Argonaute protein-containing RISC, and the other strand (also called the passenger strand) is degraded. The strand with less stable 5′ pairing ends is prioritized (Gebert and MacRae, 2019; Trabucchi and Mategot, 2020). miRNAs bind to the 3′ untranslated region (UTR) of target mRNAs and mediate their regulation at the post-transcriptional level by inhibiting translation or initiating mRNA decay (Latronico and Condorelli, 2009). Recent studies have reported that, other than gene silencing, miRNAs could stimulate transcription and translation by binding to the promoter region, 5′ UTR, or the open reading frame in target genes (Iwakawa and Tomari, 2013; Li et al., 2019). In 1993, the first animal miRNA was identified that regulates the development in *Caenorhabditis elegans* (Rosalind and Ambrost, 1993). Later, miRNAs attracted the attention of researchers (Cordes and Srivastava, 2009). A single miRNA often regulates the function of multiple mRNAs, and each mRNA can also be altered by different miRNAs, participating in precise adjustments in a complex web of cellular interactions (Leite-Moreira et al., 2013). Moreover, the two mature miRNAs originating from the different arms of a single pri-miRNA typically act on varied mRNA targets. miRNAs may contribute to cardiac homeostasis via diverse cell types, such as cardiomyocytes (CMs), cardiac fibroblasts (CFs), endothelial cells (ECs), vascular smooth muscle cells (VSMCs), and immune cells (Cordes and Srivastava, 2009; Small et al., 2010; Small and Olson, 2011; Barwari et al., 2016).

**FIGURE 1:**
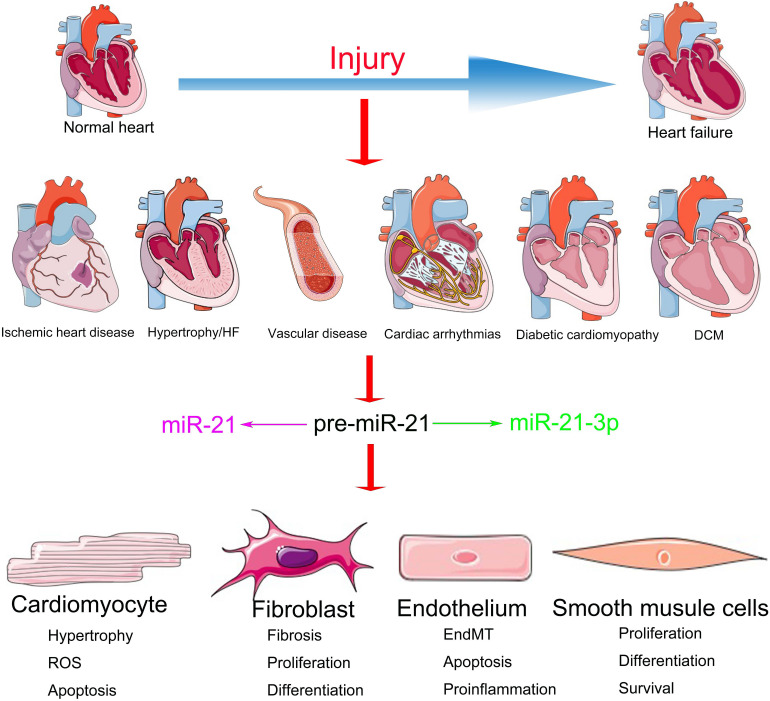
miR-21 and miR-21-3p exert various effects on diverse cell types in multiple CVDs.

The functions of miR-21 in the cardiovascular system have been extensively investigated but are still shrouded in controversy (Krichevsky and Gabriely, 2009). Although the inhibition of miR-21 in CFs via the systemic delivery of antagomirs demonstrated a significant benefit during HF (Thum et al., 2008), miR-21 knockout or knockdown of systemic expression of miR-21 with a locked nucleic acid–modified (LNA-modified) anti-miR oligonucleotide did not convert the pathological processes of HF (Patrick et al., 2010). Meanwhile, accumulating evidence suggests that the overexpression of miR-21 in the CMs demonstrates a protective role in cardiac function (Cheng et al., 2009b). This divergence might be a result of the distinct regulatory mechanisms of miR-21 in different cellular subtypes (Mishra et al., 2009) (Figure 1). Elucidating the cell type–specific functions and precise targets of miR-21 is important for potential clinical application. We have summarized the cell-specific functions and discussed the therapeutic prospects of miR-21 in CVDs in this review.

## Biology and Function of miR-21

For a long time, the human genome was originally supposed to comprise only 1% coding exons and 99% junk DNA/introns with unknown functions (Venter et al., 2001). Recently, it was recognized that the transcription of such non-coding sequences results in ncRNA rather than protein (Rosalind and Ambrost, 1993; Condorelli et al., 2014). miRNAs are short (21–25 nts) ncRNAs that are typically evolutionarily conserved in different species (Bartel, 2004). To initiate their respective functions, they bind to the respective mRNAs via the seed site (6–8 nts). By the end of 2018, according to the newly published miRbase, approximately 2000 precursors and more than 2,000 mature miRNAs are known in humans (Lucas et al., 2018). Genomic sequences located between protein-coding sequences or introns of protein-coding sequences can both encode miRNAs (Bartel, 2004). Functional and mature miRNAs are synthesized after multiple processes, including transcription, nuclear maturation, export, and cytoplasmatic processing (Beermann et al., 2016).

Various miRNAs are involved in different physiological and pathological regulations of the cardiovascular system (Small and Olson, 2011). For example, miR-1 is involved in the formation of heart tubes of chick embryos and is the most enriched miRNA in adult cardiac tissues (Darnell et al., 2006). The upregulation of its expression results in the developmental arrest at E13.5 by interacting with Hand2 (Zhao et al., 2005), which was a critical transcription factor for cardiac development (Srivastava et al., 1997). miR-133 enriched in the muscles participates in cell proliferation and cell vitality, and its knockout is linked to cardiac chamber septal impairments, serious dilatation, and embryonic death (Meder et al., 2008). Lately, miR-21 has attracted attention as one of the enriched miRNAs in the cardiovascular system for its diverse effects in cardiac function (Eva Van et al., 2006; Cheng et al., 2007; Mariko et al., 2007; Sayed et al., 2007).

The hsa-miR-21 gene is located on chromosome 17q23.2, which overlays the vacuole membrane protein 1 (VMP1) gene and is conserved. The pri-miR-21 is transcribed from the introns of VMP1 by RNA polymerase II independently (Fujita et al., 2008; Selcuklu et al., 2009). Moreover, pri-miR-21 has a conserved promoter, which is located within the intron (Ha and Kim, 2014; Adams et al., 2017; Lucas et al., 2018). Data indicate that miR-21 is regulated at the transcriptional level (Cai et al., 2004; Davis et al., 2008). For example, Fujita et al. demonstrated that a promoter sequence located at 3,770–3,337 nts upstream to the miR-21 hairpin sequence exhibits many conserved enhancer elements comprising binding sites for activator protein-1 (AP-1), CCAAT/enhancer-binding protein alpha, serum response factor, tumor protein p53, and signal transducer and activator of transcription (STAT3) (Kumarswamy et al., 2011). The miR-21 expression might be regulated post-transcriptionally. The precursor miRNA-21 (pre-miR-21) (∼70 nts hairpin structure) is processed by the endonuclease Drosha from pri-miR-21 in the nucleus (Lee et al., 2003), which might be upregulated upon extracellular stimulation (Jun et al., 2009). Subsequently, pre-miR-21 is exported by Exportin 5 and processed by Dicer to release mature hsa-miR-21 (also known as hsa-miR-21-5p from the 5p arm of the pre-miR-21, which is the biologically dominant arm) and hsa-miR-21-3p (formerly named hsa-miR-21^∗^ from the 3p arm of the pre-miR-21, which was previously considered less abundant than hsa-miR-21-5p) in the cytoplasm (Kumarswamy et al., 2011). Both hsa-miR-21 and hsa-miR-21-3p exhibit important functions in the cardiovascular system by targeting different mRNAs (Thum et al., 2008; Roy et al., 2009; Yan et al., 2015). In particular, the functions of miR-21 in CVDs are of vital importance (Zhang, 2008).

MiR-21 is typically abundant in the dominating cellular subtypes of the cardiovascular system (Zhang, 2008), including CMs (Cheng et al., 2007), ECs (Suarez et al., 2007), and VSMCs (Ji et al., 2007) and especially CFs (Roy et al., 2009). Previous studies have demonstrated the important characteristics of miR-21 in the cardiovascular system by experiments with gain- and loss-of-function mutations (Eva Van et al., 2006; Cheng et al., 2007; Mariko et al., 2007; Sayed et al., 2007; Thum et al., 2007b). However, the expression patterns and functions of miR-21 reported in various CVDs are controversial (Supplementary Table 1). For instance, researchers observed that during HF, the expression of miR-21 is particularly upregulated in CFs, which, in turn, activates the extracellular signal-regulated kinase-mitogen activated protein kinase (ERK-MAPK) pathway by inhibiting sprouty homolog 1 (SPRY1) protein. The increased expression of miR-21 promotes cardiac remodeling by improving the viability of CF and the accumulation of hypertrophy-inducing factors. Furthermore, the inhibition of miR-21 in mice resulted in the inactivation of the ERK-MAPK pathway and the prevention of CM hypertrophy along with CF activation (Thum et al., 2008). On the contrary, transgenic mice with miR-21 overexpression demonstrated a reduction in the infarct area and CF fibrosis as well as the downregulation of phosphatase and tensin homolog (PTEN) protein and Fas ligand (FasL) in ischemic diseases (Sayed et al., 2010). These contradictions might be a result of the abundance and diverse targets of miR-21 in different cell types, which ultimately leads to miscellaneous effects on the cardiac function during different stages and processes of CVDs (Figure 1).

### Ischemic Heart Disease: The Role of miR-21 in CMs and CFs

According to the CVD epidemiological update in Europe in 2016 (Townsend et al., 2016), plaque damage after coronary and sequent hypoxia results in AMI, which was the major cause of morbidity and mortality. CM death (Francis Stuart et al., 2016; DeLeon-Pennell et al., 2017; Frangogiannis, 2017), the activation of EC and CF, and necrotic cell removal are involved in the progression of ischemia (Ma et al., 2014). Despite the availability of therapeutic approaches, AMI is still associated with high rates of acute death and long-term complications, such as HF. Early diagnosis and intervention are pivotal in reducing the damage caused by AMI. Currently, the most frequently used diagnostic biomarker is a highly sensitive group of cardiac proteins known as troponins (Roffi et al., 2016). However, these markers are not without limitations. Elevated troponin levels could be observed in congestive HF patients or individuals with long-term kidney diseases as well (Rubini Gimenez et al., 2014).

Recently, miRNAs were evaluated as potential markers of AMI (Fiedler and Thum, 2013). Corsten et al. (2010) investigated 32 citrate plasma samples from patients with AMI (obtained at the time of mechanical reperfusion) and 36 plasma samples from patients with atypical chest pain and positive stress testing; however, normal coronary angiograms, using RT-PCR arrays, helped in observing that the AMI patients demonstrated higher levels of circulating miR-21 as compared to those of the control group. Oerlemans et al. (2012) also used RT-PCR arrays to explore the potential diagnostic value of circulating microRNAs as novel early biomarkers in 332 suspected acute coronary syndrome (ACS) patients (Oerlemans et al., 2012). The serum samples were collected before treatment interventions. They observed that miR-21 in combination with miR-1, miR-499, and high-sensitive troponin T (hs-cTnT) performed well in diagnosing ACS (AUC = 0.90, *n* = 106). Elevated miR-21 could be observed even in patients with ACS who tested negative for hs-cTnT initially or showed symptom onset in <3 h (Oerlemans et al., 2012). However, using TaqMan PCR arrays, Liebetrau et al. (2013) demonstrated that miR-21 did not increase in patients undergoing transcoronary ablation of septal hypertrophy (TASH), which was used as a model for mimicking AMI (13 males and 8 females with the average age of 59.0 ± 13.29 years). In this study, venous blood samples were collected before and at different time points after the induction of MI for the determination of miRNAs. The variation in the conclusions derived in these studies may be due to the differences in the methods of sample collection (when and how the samples were collected), clinical and demographic parameters (age, sex, and medical history), and detection methods (RNA isolation and PCR arrays).

In addition to the possible utilization of the circulating miR-21 as a biomarker for AMI, its intracellular effects might help improve the cardiac function post-AMI. Roy et al. (2009) first reported that ischemia reperfusion markedly induced the expression of miR-21 in the infarct region of the heart. Increased miR-21 resulted in fibroblast survival and triggered fibrotic infarct remodeling by the inhibition of PTEN in CFs (Roy et al., 2009). Another study also indicated that miR-21 mediated the activation of fibroblasts caused by transforming growth factor-β (TGF-β) by targeting Jagged1 (Bronnum et al., 2013) and SMAD family member 7 (SMAD7) (Yuan et al., 2017). However, Dong et al. (2009) demonstrated the upregulation and downregulation of miR-21 expression in the infarct and border region, respectively, in the AMI rat model. Overexpression of miR-21 protected the cultured CMs against apoptosis by regulating programmed cell death 4 (PDCD4) and the AP-1 pathway (Dong et al., 2009). Sayed et al. (2010) showed that protein kinase B (AKT) upregulated the miR-21 expression. miR-21 transgenic mice exhibited a smaller infarct area and suppressed HF by downregulating the enhanced PTEN and FasL expression in the ischemic heart in CMs (Sayed et al., 2010). miR-21 increased cardiac fibrosis, which led to increased cardiac function in the CFs. It also reduced apoptosis and demonstrated a protective role in the cardiac function in CMs.

Interestingly, exosomal miR-21 participated in angiogenesis (Wang et al., 2017), cell proliferation (Xiao et al., 2016), cardiac remodeling, and metabolic regulation via the paracrine signaling networks in the target cells as well (Luther et al., 2018). For example, Chen et al. (2019) demonstrated that the knockout of exosomal miR-21 in CMs cultured with an oxygen-glucose–deprived media increased the ROS-induced apoptosis in CMs by targeting PDCD4 and decreased the activation of CFs and angiogenesis mediated by ECs, demonstrating the interaction between CMs and other cell types in the heart.

**FIGURE 2:**
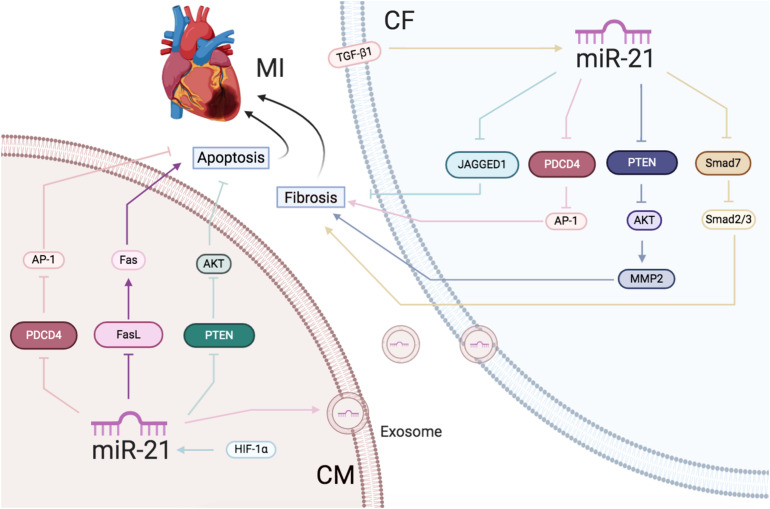
Ischemic heart disease: the roles of miR-21 in CMs and CFs.Reported major signals in CMs. Pink lines: miR-21 decreases PDCD4/AP-1 to inhibit apoptosis. Blue lines: HIF-1α-induced miR-21 downregulates PTEN/AKT to reduce apoptosis. Purple lines: miR-21 decreases FasL/Fas to reduce apoptosis. Reported major signals in CFs. Yellow lines: TGF-β-induced miR-21 downregulates Smad7 and activates Smad2/3 to increase fibrosis. Blue lines: miR-21 inhibits JAGGED1 to increase fibrosis. Gray lines: miR-21 inhibits PTEN/AKT/MMP2 to increase fibrosis. Pink lines: exsomal miR-21 in CMs inhibits PDCD4/AP1 pathway to promote CFs fibrosis.

In conclusion, increased miR-21 expression caused by AMI protected the CMs from apoptosis along with enhancing the activation of CFs (Figure 2).

### Cardiac Hypertrophy and Heart Failure: The Roles of miR-21 in CMs, CFs, and ECs

Pathological cardiac hypertrophy is primarily caused by chronic hemodynamic overload, ischemic injury, altered metabolism, or neuroendocrine activity (Barry et al., 2008). Loss of CM and excessive deposition of the extracellular matrix are major contributors to the development of cardiac hypertrophy to HF (Creemers and Pinto, 2010). Preventing cardiac hypertrophy and fibrosis is of vital importance in the prevention of HF (Frangogiannis, 2012).

Thum et al. (2007b) showed that the altered expression of miRNAs and mRNAs in the human fetal heart was closely associated with HF and the overexpression of several fetal miRNAs in CMs, including miR-21, which led to cellular hypertrophy and alternated gene expression, giving rise to the conditions mimicking HF. Eva Van et al. (2006) discovered that miR-21 expression increased in the hypertrophic heart but did not change in patients with HF. Further, by using animal models, Cheng et al. (2007) and Mariko et al. (2007) reported the upregulation of miR-21 expression with the progression of hypertrophy in the heart and had a negative effect with respect to the size of CM. The varied patterns of miR-21 expression in these studies might be due to the variation in the sampling intervals (the stage of hypertrophy and heart failure) and the medical history of patients (the cause of heart failure should be taken into account). Moreover, the sources influencing miR-21 expression are complex. The expression of miR-21 might be varied in multiple cell types in the heart during different stages of disease progression. Indeed, several studies have emphasized different influences of miR-21 on CMs and non-CMs, especially CFs. Thum et al. (2008) showed that the expression of miR-21 in CMs was low in the base state. It increased selectively in CFs rather than CMs during heart failure. miR-21 activates the ERK-MAPK pathway via the inhibition of SPRY1 and mediates the structural and functional deterioration of cardiac function (Thum et al., 2008). In addition, the activation of the transcription factor AP-1 and subsequent miR-21 expression mediated Ang II-induced cardiac fibrosis. miR-21 led to the inhibition of the antiapoptosis and antifibrosis targets PTEN and SMAD7, ultimately leading to the proliferation of CFs (Lorenzen et al., 2015). The administration of antagomir that acts against miR-21 in mice with left ventricular pressure overload attenuated the endothelial-to-mesenchymal transition in ECs *in vivo* (Kumarswamy et al., 2012).

Exosomes replicated the redeeming function of the host cells in target cells, partly by paracrine action. The composition and biological activities of exosomes primarily depend on the cells secreting the exosomes. Qiao et al. (2019) observed that the exosomes in patients with HF derived by explant heart tissue demonstrated decreased CM proliferation and angiogenesis mediated by ECs leading to cardiac dysfunction. miR-21 overexpression in the exosomes suppressed PTEN/AKT signaling to improve CM vitality and angiogenesis (Qiao et al., 2019). The fate of miR-21-3p has also garnered interest due to its influence on the progression of cardiac hypertrophy to HF (Duygu and Da Costa Martins, 2015). Deep RNA sequencing demonstrated increased expression of miR-21-3p during HF in humans (Yang et al., 2014). Exosomal miR-21-3p in CFs could induce CM hypertrophy by communications between the two cell types with paracrine signaling (Bang et al., 2014). Meanwhile, our group identified that miR-21-3p exerted an antihypertrophic effect on CMs by targeting HDAC8 and observed reduced expression of miR-21-3p in hearts after 2 weeks, which was increased after 4 weeks following transverse aortic constriction (TAC) (Yan et al., 2015). The contrasting roles of miR-21-3p might be attributed to different cell types. Patients often showed abnormalities in cardiac structure and function with high blood pressure, such as left ventricular hypertrophy and HF (Lip et al., 2000). In our previous studies, we demonstrated that the systemic delivery of miR-21 in animals decreased the blood pressure and improved cardiac hypertrophy in the spontaneously hypertensive rat (SHR) by upregulating mitochondrial cytochrome B (CYTB). The miR-21 expression in hypertensive patients was increased with the rise in blood pressure (Li et al., 2016).

**FIGURE 3:**
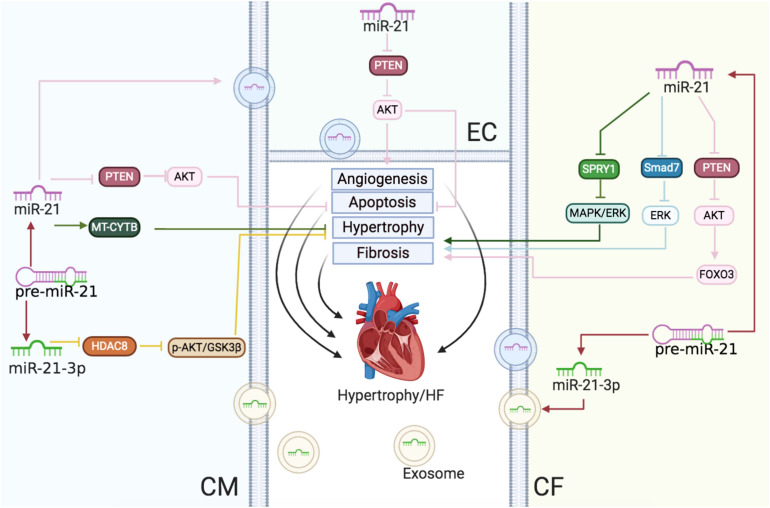
Cardiac hypertrophy and HF: the roles of miR-21/miR-21-3p in CMs, CFs, and ECs. Reported major signals in CMs. Green lines: in hypertension, miR-21 enhances MT-CYTB mRNA translation in the mitochondria to inhibit hypertrophy. Pink lines: in HF, exosomal miR-21 suppresses the PTEN/AKT signaling to inhibit apoptosis. Yellow lines: miR-21-3p downregulates the HDAC8/p-AKT/GSK3β pathway to decrease hypertrophy. Reported major signals in CFs. Pink lines: miR-21 downregulates PTEN and enhances the AKT/FOXO3 pathway to induce fibrosis. Blue lines: miR-21 inhibits the SMAD7/ERK pathway to induce fibrosis. Green lines: miR-21 downregulates SPRY1/ERK/MAPK to increase fibrosis. Reported major signals in ECs. Pink lines: exosomal miR-21 from CMs downregulates PTEN to decrease EC apoptosis and induce angiogenesis.

In conclusion, during the progress of cardiac hypertrophy to HF, upregulated miR-21 reduced CM sizes but also mediated cardiac fibrosis (Figure 3).

### Vascular Disease: The Role of miR-21 in ECs and VSMCs

Injuries or alterations would promote EC fragility or migration and VSMC dedifferentiation, migration, and proliferation in vessels (Leite-Moreira et al., 2013). Cellular phenotypic transformation accounted for the progress of proliferative diseases, including atherosclerosis, in-stent restenosis, and hypertension (Cahill and Redmond, 2016). This further led to a sudden heart attack and vessel disease (Small et al., 2010). Recently, miRNAs, including miR-21, were proposed to modulate their expression in response to the effector molecules in endothelial and VSMCs (Cheng et al., 2009a; Cordes et al., 2009; Davis et al., 2009; Xin et al., 2009).

The abdominal aortic aneurysm (AAA) leads to high morbidity and mortality rates worldwide (Creager et al., 2004). Maegdefessel et al. (2012) observed that miR-21 was upregulated and its target PTEN was downregulated in human aortic samples, which were obtained from patients with AAA who underwent surgical repair of the augmentative abdominal aorta (57–68 mm). miR-21 was also found to be upregulated primarily in human arteriosclerosis obliterans (ASO) samples. HIF-1α induced miR-21 targeted tropomyosin 1 (TPM1) to promote VSMC proliferation and migration during ASO (Wang et al., 2011). miR-21 was shown to be present in abundance in the failed human transplant similar to that in the animal transplant models (McDonald et al., 2013). miR-21 knockdown derepressed PTEN to reduce the neointima formation and phenotypic transition in VSMCs and CFs in a rat balloon surgery (Ji et al., 2007). According to Davis et al. (2008), miR-21 affected TGF-β and bone morphogenetic protein (BMP) expression and slowed down human VSMCs, leading to a contractile phenotype by downregulating PDCD4. Furthermore, TGF-β and the BMP pathway accelerated the cleavage of pri-miR-21 into pre-miR-21 by Drosha complex, thus elevating the mature miR-21 levels post transcription (Davis et al., 2008).

Interestingly, for the first time, our studies observed that amlodipine-activated AKT2 not only increased SP1 translocation into the nucleus, but also the cooperative binding to the miR-21 promoter, which finally targeted PDCD4 to change the phenotype of VSMCs (Fang et al., 2019). Meanwhile, we also demonstrated that miR-21-3p levels were significantly declined in both hypertensive patients and SHR plasma. miR-21-3p upregulation caused a sustained attenuation of hypertension with a significant reduction in the destruction of the target organs, including arterial and kidney fibrosis as well as cardiac hypertrophy and fibrosis in SHRs by suppressing adrenal α2β-adrenergic receptor (ADRA2B) in the arteries (Wang F. et al., 2018).

miR-21 targeted RhoB reduced the EC proliferation and migration and decreased the ability to form tubules, thereby leading to negative regulation of angiogenesis (Capogrossi et al., 2011). However, miR-21 promoted endothelial dysfunction and atherosclerotic lesion development under shear stress (SS) (Davies, 2009). Zhou et al. (2011) verified that oscillatory SS-induced miR-21 repressed PPARα, which, in turn, reduced the inhibition of the AP-1 pathway by PPARα. Positive feedback was observed increasing the transcription of miR-21 and inflammation in ECs, suggesting that the inhibition of miR-21 might be a key treatment approach for regulating EC dysfunction (Zhou et al., 2011). In previous studies, miR-21 increased fivefold in human umbilical vein endothelial cells (HUVECs) subjected to unidirectional SS for 24 h and increased miR-21 inhibited PTEN to enhance nitric oxide (NO) production and EC viability (Weber et al., 2010).

**FIGURE 4:**
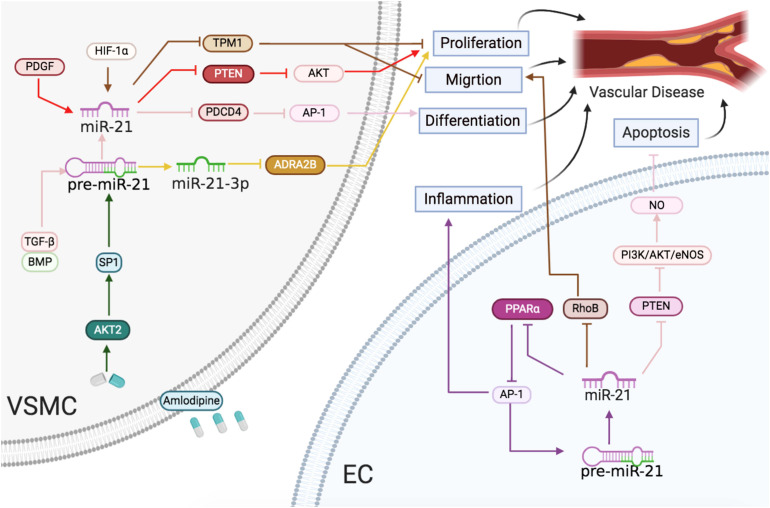
Vascular diseases: the roles of miR-21/miR-21-3p in ECs and VSMCs. Reported major signals in ECs. Brown lines: miR-21 represses RhoB to reduce migration. Purple lines: miR-21 downregulates PPARα and activates AP-1 to accelerate inflammatory response. Pink lines: miR-21 blocks PTEN and activates the PI3K/AKT/eNOS pathway to enhance NO level and decrease apoptosis. Reported major signals in VSMCs. Red lines: in neointima formation, PDGF-induced miR-21 inhibits PTEN to induce proliferation. Brown line: in arteriosclerosis obliterans, HIF-1α-induced miR-21 downregulates TPM1 to induce proliferation and migration. Pink line: TGF-β/BMP-induced miR-21 downregulates the PDCD4/AP1 pathway to promote differentiation. Green line: in hypertension, the amlodipine-activated AKT2/SP1 pathway to induce miR-21 and upregulated miR-21 downregulates the PDCD4/AP1 pathway to promote differentiation. Yellow line: in hypertension, miR-21-3p suppresses ADRA2B to reduce proliferation.

In summary, vessel injuries inducing miR-21 expression resulted in VSMC and EC proliferation, migration, and differentiation, leading to atherosclerosis, hypertension, and restenosis (Figure 4).

### Cardiac Arrhythmias: The Role of miR-21 in CMs and CFs

Cardiac arrhythmias are the abnormalities or perturbations in the normal activation or the rhythms of the heart myocardium. Various factors contribute to cardiac arrhythmias, including ischemia, electrolyte disturbance, scarring, aging, and certain medications (Fu, 2015). These factors result in CM hypertrophy, abnormal electrical activity, and cell death, which might induce fibrosis. Indeed, fibrosis is a decisive factor of myocardial heterogeneity and a major marker for HF, which augmented the trend for reentry arrhythmias and diastolic stiffness (Swynghedauw, 1999). miR-21 is also essential in the regulation of CM and CF phenotype in cardiac arrhythmias.

Upregulated miR-21 in the left atria leads to the downregulation of its downstream molecule SPRY1 in atrial fibrillation (AF) patients. A previous study demonstrated that increased miR-21 expression activated Rac1-GTPase, LOX, and CTGF, which subsequently augmented ECM deposition and activated Drosha and Dicer (Adam et al., 2012). According to Barana et al. (2014), increased miR-21 induced by chronic AF downregulated L-type calcium current flow, which acts as a signal of electrical disorders in detached human atrial CMs. Further, Huang et al. (2016) revealed that heart surgery initiated a neoteric reciprocal flow of STAT3 and miR-21. Inhibition of miR-21 repressed p-STAT3 in CFs, which relieved AF and reduced atrial conduction disorders and predisposition in AF. In contrast, AF patients exhibited decreased miR-21 expression in plasma, and AF status representing the severity of AF (paroxysmal vs. persistent) was proportional to miR-21 expression. Interestingly, miR-21 expression increased 1 month after AF disappearance (McManus et al., 2015).

**FIGURE 5:**
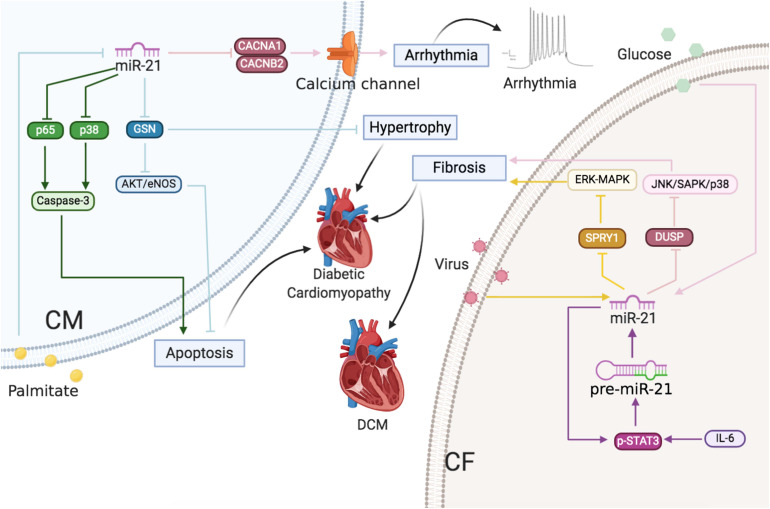
Cardiac arrhythmias and cardiomyopathy: The roles of miR-21 in CMs and CFs. Reported major signals in CMs. Pink line: in cardiac arrhythmias, miR-21 downregulates Ca^2+^ channel subunits CACNA1C and CACNB2 to decrease *I*_*Ca,L*_ density. Green line: in diabetic cardiomyopathy, palmitate decreases miR-21 and then miR-21 inhibits p65 and p38 to decrease apoptosis. Blue line: in diabetic cardiomyopathy, palmitate decreases miR-21 and then miR-21 inhibits GSN to decrease hypertrophy and apoptosis. Reported major signals in CFs. Yellow line: in DCM, virus-induced miR-21 inhibits SPRY1 to induce fibrosis. Purple line: in AF, IL-6-enhanced miR-21 via p-STAT3 positive feedback, and miR-21 inhibits SPRY1/ERK/MAPK to induce fibrosis. Pink line: in diabetic cardiomyopathy, glucose-induced miR-21 decreases DUSP8 to increase fibrosis.

Collectively, the aforementioned studies demonstrated that increased miR-21 expression during the cardiac arrhythmias not only induced CM electrical remodeling but also enhanced CF activation, leading to severe fibrosis (Figure 5).

### Cardiomyopathy: The Role of miR-21 in CMs and CFs

Diabetic cardiomyopathy is characterized by early diastolic dysfunction, final systolic dysfunction, cardiac hypertrophy, and ventricular dilation in the heart and leads to HF (Tabak et al., 2012; Guo and Nair, 2017). High glucose levels and fatty acid utilization disorders lead to apoptosis of CMs (Tziomalos et al., 2010), fibroblast activation, and EC dysfunction (Bauersachs and Thum, 2007; Thum et al., 2007a). In recent studies, miR-21 was demonstrated to act as a potential regulator for diabetic cardiomyopathy. Most studies show a decline in miR-21 levels in the plasma of diabetic patients (Zampetaki et al., 2010; Jansen et al., 2016; Giannella et al., 2017). Moreover, bariatric surgery or exercises might restore the miR-21 level in the circulation of diabetic/prediabetic patients (Villard and Marchand, 2015; Lew et al., 2017). Furthermore, miR-21 was overexpressed in diabetes, diabetes with coronary artery disorders, and diabetes with acute HF, particularly in diabetes with acute HF according to the recent statistics. Measurement of miR-21 levels might be beneficial in predicting the occurrence of acute HF in symptomless diabetics (Al-Hayali et al., 2019).

The miR-21 expression is particularly elevated in high-glucose-cultured CFs, which results in increased collagen cross-link and cardiac fibrosis via Jun amino-terminal kinases/stress-activated protein kinases (JNK/SAPK) and p38 by regulating dual specific phosphatase 8 (DUSP8) (Liu et al., 2014). On the contrary, Zhou et al. (2018) reported that miR-21 was downregulated in palmitate-treated CMs. miR-21 protected CMs from apoptosis through inhibiting p65 and p-p38 expression. Our group also observed downregulated miR-21 in palmitate-treated CMs and db/db mice, which resulted in diabetes-induced diastolic dysfunction by increased gelsolin (GSN) levels and reduced NO production via Akt-eNOS-NO signaling (Dai et al., 2018). The reason for the varied observations might be attributed to the fact that, although high glucose and high fat contribute to the etiology of diabetic cardiomyopathy, different sources have varied effects on miR-21 expression, which could exert diverse functions in multiple cell types. Liu et al. (2014) observed that high glucose increased the levels of miR-21, whereas Dai et al. (2018) and Zhou et al. (2018) observed that miR-21 expression decreased in response to palmitate. In addition, Liu et al. (2014) observed that miR-21 increased collagen cross-link in CFs and cardiac fibrosis, whereas Dai et al. (2018) and Zhou et al. (2018) found that miR-21 exerted a protective effect in CMs.

Chronic inflammation of the heart tissue caused by the viral infection (principally by the coxsackievirus, HIV, and adenovirus hepatitis virus) is termed viral myocarditis (VMC) (Pollack et al., 2015; Fung et al., 2016; Wang Y. et al., 2018). Only 60% of pediatric patients with acute myocarditis survived for 10 years (Towbin et al., 2006), and 9% of patients suffered from dilated cardiomyopathy (DCM), and 12% of young adults died in a short interval following the onset of VMC (Fabre and Sheppard, 2006). DCM characterized by ventricular dilatation and systolic dysfunction would lead to arrhythmia and HF (Maisch et al., 1996; Arthur and Feldman, 2000). Multiple mechanisms have been implicated in the progression from VMC to DCM (Cooper et al., 2010), including direct viral injuries on CMs, cardiac fibrosis, and inflammatory responses (Xu et al., 2014). miR-21 expression increased in the heart of acute VMC patients as well as in that of coxsackie B3 (CVB3)-infected VMC mice (Corsten et al., 2012; Fung et al., 2016). More importantly, upregulated miR-21 promoted cardiac fibrosis during the progression of VCM to DCM by inhibiting SPRY1 and enhancing the MAPK signaling pathway (Xu et al., 2014). *In vivo* silencing of miR-21 in mice with VMC might reduce inflammatory lesions, suppress T helper 17 cell differentiation, and rescue heart function (Liu et al., 2013).

In conclusion, high glucose level and viral injuries induced miR-21-enhanced cardiac fibrosis, whereas reduced miR-21 expression caused by palmitate led to CM apoptosis and cardiac hypertrophy (Figure 5).

## Regulation of miRNA-21 in CVDs

**FIGURE 6:**
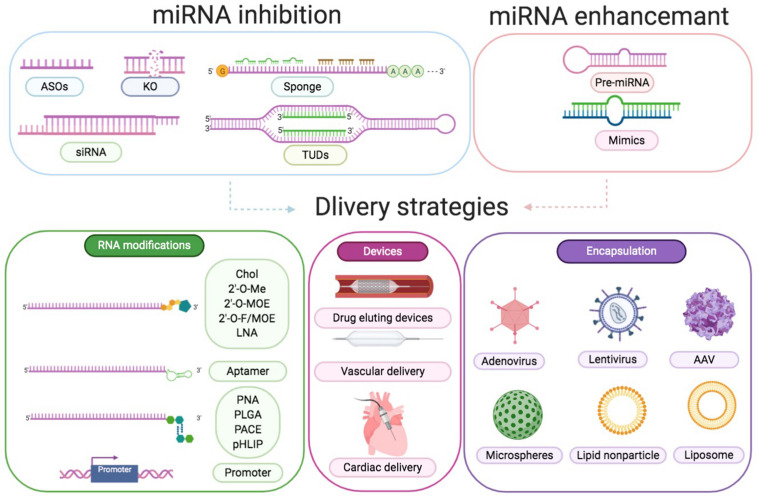
Approaches to modulate miRNA function. Downregulation of a certain miRNA preferentially could be achieved by utilization of specific ASOs, siRNA, miRNA sponges, TUDs, or by genetic knockout. Overexpression of a specific miRNA could be achieved by using miRNA mimics or pre-miRNA expression systems. Delivery strategies are RNA modifications, devices, and encapsulation. RNA modifications include conjugation to cholesterol (Chol), 2′-*O*-methyl (MOE), LNA, aptamer, peptide nucleic acid (PNA), poly lactic-co-glycolic acid (PLGA), poly amine-co-ester terpolymer (PACE), pH low insertion peptide (pHLIP), and cell-specific promoter. Devices include drug eluting devices, vascular delivery, and cardiac delivery. Encapsulation includes adenovirus, lentivirus, AAV, microspheres, lipid nanoparticles, and liposomes.

As highlighted above, miR-21 participates in infarction injuries, cardiac remodeling, atherosclerosis, arrhythmias, and cardiomyopathy, which are caused by infection or metabolic disorders (Thum et al., 2008; Thum, 2012, 2014; van Rooij and Olson, 2012; Fiedler and Thum, 2013; Li et al., 2016; Dai et al., 2018; Zhou et al., 2018). This indicates its potential as a clinical therapeutic target. However, miR-based treatments have challenges associated with it. At present, two major procedures are designed to alter miRNA expression. To decrease the expression of a specific miRNA, specific antisense oligonucleotides (ASOs), small interfering RNA (siRNA), miRNA sponges could be typically utilized. Genetic knockout and synthetic miRNA mimics or pre-miRNA in viral vehicles are used to enhance a specific miRNA level (Figure 6).

Antagomirs and LNA anti-miRs are the most prominent examples of ASOs, which perfectly match to the target miRNA in a complementary fashion to block the inhibitory function of miRNAs (Obad et al., 2011). Thum et al. (2008) reported that cholesterol-modified antagomiR-21 protected against cardiac hypertrophy and fibrosis by reacting to TAC-induced pressure overload. However, Patrick et al. showed that LNA-antimiR-21 and miR-21 knockout decreased the levels of miR-21 but did not alter the pathological process associated with pressure overload or other stimulations (Eva Van et al., 2006). Subsequently, Thum et al. (2011) compared the efficiency of the three different anti-miRs and discovered that treatment with two long antagomirs reduced cardiac fibrosis and hypertrophy, whereas tiny LNAs did not exert any beneficial effect. Recently, despite the improvements in anti-miRs [selenomethylene LNAs (Nahar et al., 2016), small RNA zippers (Meng et al., 2017), and peptide nucleic acids (Paulasova and Pellestor, 2004)], no more approaches are published exploring the potential application for CVD treatment. SiRNAs, other inhibitors in RNA duplexes, are combined with the loop sequence of the miRNA or a particular sequence of mRNA to protect the respective miRNA targets (Behlke, 2008). The third miRNA inhibitors, known as miRNA sponges, prevented the action of a specific miRNA by sponging to miRNA seed regions or the mRNA sequence of the respective miRNA target (Messina et al., 2016). However, the proper dosage of siRNA and miRNA sponges to counter certain endogenous miRNA concentrations is difficult to define. Furthermore, Cheng et al. (2009b) have designed and added tough decoys (TuD)-miR-21 into lentiviral vehicles for long-term suppression of miR-21.

The induction of miRNA levels is even more ambitious compared to miRNA inhibition. Double-stranded RNA fragments acting as miRNA mimicked the endogenous miRNAs and replaced or enhanced miRNA density in tissues (Olson, 2014). Mature miR-21 mimics are widely used to overexpress miR-21 *in vitro* and can be easily purchased from the industry (Liang et al., 2012; Bang et al., 2014). Although high concentrations of miRNA mimics could achieve a significant increase in target miRNAs (Bader et al., 2010), miRNA mimics might also result in unanticipated side effects. Pre-miR-21 in lentivirus vector was proven to efficiently overexpress miR-21 (Wang J. et al., 2018), and could specifically be overexpressed in CM by combining with the mouse myosin-6 promoter (Thum et al., 2008). However, these viral vectors overexpressing the precursor sequence might finally result in a complex effect by synthesizing both miR-21 and miR-21-3p (Schober et al., 2014).

In principle, there are two prime goals, namely reduction of the dose and the risk of toxicity and sequence-specific side effects. To improve cellular absorption and decrease dose-dependent toxicities, delivery strategies consisting of altering the structure to obtain resistance against RNases, adding cholesterol, and decreasing the immunoreaction and off-target effects should be concerned (Lucas et al., 2018). Obtaining tissue or cell-type specificity is another obstacle to overcome. Compared with miR-21 inhibition, anti-21-coated stents confined miR-21 in the local circulatory system and exerted fewer side effects according to Wang et al. (2015). AAV serotype 9 viruses demonstrated a preference for the heart with a specific dose, which could be applied to delivery miRNAs, shRNAs, and mRNAs in animals (Care et al., 2007; Li et al., 2009; Eulalio et al., 2012; Karakikes et al., 2013). Ramanujam et al. (2016) generated AAV9 and Moloney murine leukemia virus vectors for the deletion of miR-21 in the CM- and non-myocyte-specific manner in chronic left ventricular pressure overload models. Our group tried AAV9 and CM-specific promoter troponin T to specifically express miR-21 in CMs and proved that miR-21 demonstrated a protective role in CMs during diabetes (Dai et al., 2018). Furthermore, adding specific promoters, such as transcription factor 21 and fibroblast specific protein 1 (FSP1), to ECs and CFs, respectively, would allow specific effector molecules to be expressed (Song et al., 2012). Only miR-21 knockout mice have been preferred (Patrick et al., 2010; Lu et al., 2011) owing to the limitations of promoter strength (Lindsley et al., 2007; Qian et al., 2012) and affinity with cells and organs (Acharya et al., 2011; Kong et al., 2013). For example, β-galactosidase activity under the control of FSP1 was observed in many but not all CFs and certain endocardial and ECs as previously reported. Several novel approaches demonstrating miRNA-specific delivery have been published so far. In addition to assembled AAV particles with large polypeptides coupled to the surface and aptamer-linked miRNAs (Muik et al., 2017), new technologies, including nanoparticles and advanced biomedical materials, have been developed for the transport of particular molecules to the desired location. miR-21 mimics could increase perfusion and cell viability while reducing cardiac remodeling post-MI in the border area by delivering nanoparticles to macrophages, demonstrating a new treatment strategy (Bejerano et al., 2018). Furthermore, Alexy et al. (2014) reported that miR-21 was reduced in microparticles (MPs) released by ECs in response to tumor necrosis factor α (TNF-α). They also observed that Rho-associated coiled-coils containing protein kinase-dependent, miRNA-rich MPs could transfer their contents effectively and were antiapoptotic, whereas caspase-dependent, miRNA-poor MPs were proapoptotic. The aforementioned results demonstrated an underlying application of cell-to-cell interaction by packaging miRNAs in MPs (Alexy et al., 2014). Although the novel therapeutics described above have not been applied for clinical use, they demonstrate a great promise.

## Discussion

The influence of miR-21 on the cardiovascular system is not negligible. However, several controversies have been associated with it. Circulating miR-21 are potential biomarkers for CVDs, but their levels have been inconsistent in the published reports. First, the blood sample used for detection might not be subjected to standardized preparation methods, and the plasma sample might differ from the serum sample. The miR-21 levels are not constant during the course of a given disease. For example, miR-21 levels in the acute period of AMI (upregulated) would be different from those obtained after successful PCI therapy (downregulated). Second, the number of participants as well as their past medical history, age, gender, and race may also result in differential expression levels of circulating miR-21. A multicenter independent study with a large cohort will contribute to the stability and reliability of the discovery data (Small and Olson, 2011). In addition, the source cell type of miR-21 in the circulating system is unknown, and miR-21 might be secreted from diverse cell types under different stimuli. miR-21 expression in the tissues involves the same contentions. For example, the expression of miR-21 varies with the stages of AMI (Gu et al., 2015) and localization of the cell type within the heart (Roy et al., 2009). Therefore, the expression pattern of miR-21 in the heart may change. Recently, many researchers have concentrated on the cellular-specific expression of miR-21.

Moreover, studies have observed that miR-21 expression is important in other diseases, such as cancer (Bonci, 2010), kidney fibrosis (Chau et al., 2012), metabolic syndrome (Calo et al., 2016), and asthma (Kim et al., 2017). The targets of miR-21 in CVDs might also participate in the etiology of such diseases. For example, PTEN, which is considered as a cancer suppressor, also plays a role in CMs, CFs, and ECs by influencing cell apoptosis and proliferation. miR-21-dependent PPARα downregulation could affect fatty acid oxidation and trigger steatosis in hepatocytes (Loyer et al., 2016) as well as mediate kidney injury and fibrosis in epithelial cells (Chau et al., 2012). Meanwhile, miR-21 targeted PPARα resulted in increased inflammation owing to the ECs in the heart. Thus, when we discuss the function of miR-21, the cell types and specific pathological status must be carefully considered. Current research has also demonstrated that miR-21 might serve as a potential biomarker for the diagnosis, prognosis, and prediction of multiple diseases (Ramachandran et al., 2013; Bautista-Sanchez et al., 2020). According to Qu et al. (2017), serum miR-21 was especially upregulated in patients with pancreatic cancer with a sensitivity of 0.77 and specificity of 0.8104 in 56 pancreatic cancer patients from six medical centers in China. Ramachandran et al. (2013) have indicated that miR-21 might be a sensitive (and non-invasive) indicator of kidney damage in pooled urine samples from 6 patients with acute kidney injury (AKI) and 6 healthy controls as demonstrated by miRNA PCR array (miRBase version 18, containing 1,809 miRNAs; Qiagen). This increases the complexity in the assessment of the roles of miR-21 in CVDs. We should carefully include proper negative controls while investigating the role of miR-21 in CVD patients. Further, miR-21 expression should be combined with the results of the evaluation of clinical symptoms and lab examination of the patients for a comprehensive diagnosis of the disease.

Because the function of miR-21 is mediated by its target genes, it is important to identify its direct targets. Studies have reported various targets of miR-21 not only in different cell types but also in the same cells. We speculated that one of the reasons might be the fact that various bioinformatic tools using different algorithms were employed in different studies to predict the targets of hsa-miR-21 (Table 1). For example, we searched the updated miRTarBase and observed that both miR-21-5p and miR-21-3p exhibited binding sites in the 3′ UTR region of PTEN. Meanwhile, PTEN was predicted as the target of miR-21-5p by using a modified bioinformatics approach proposed by Doench and Sharp (2004) (Kiriakidou, 2004; Meng et al., 2006; Doench et al., 2005). However, PTEN was not predicted as the biological target of hsa-miR-21 or miR-21-3p by the latest Targetscan (Ver 7.2) approach. The algorithm of a certain bioinformatics website might be constantly updated (Costa et al., 2020). For example, PTEN was not predicted as the target of miR-21-3p by the latest Targetscan (Ver 7.2), but it was also predicted as the target of hsa-miR-21-3p by using TargetScan (Ver 3.1), miRWalk and miRbase (Zhu et al., 2019). Furthermore, conventionally, miRNAs always bind to the 3′ UTR of their target mRNAs. However, in recent years, it has been reported that miRNAs could also bind to the CDS, promoter, and 5′ UTR regions of the target genes (Iwakawa and Tomari, 2013; Li et al., 2019). Several binding sites of hsa-miR-21 in the CDS, promoter, and 5′ UTR regions of PTEN were also predicted by the BiBiServ using an RNA–RNA hybrid. Most algorithms use seed regions for Watson–Crick matching of the target, but the secondary structure of the mRNA may also contribute to the binding (Didiano and Hobert, 2006). Therefore, considering the complexity in the prediction of direct targets of miRNAs, biological experiments are essential to validate the bioinformatic predictions. Studies have verified PTEN as the direct target of hsa-miR-21-5p and hsa-miR-21-3p using biological experimental methods, such as luciferase reporter assay, western blot, and qRT-PCR (Sayed et al., 2010; Qiao et al., 2019; Zhu et al., 2019). Considering the comprehensive signaling network, it is possible that PTEN is not only a direct target of miR-21, but is also regulated at a “second” level by a product of a target mRNA, which makes the identification process more complex.

**TABLE 1:** Useful web links for miRNA target prediction.

**miRNA targets within 3′ UTR**	
PicTar	https://pictar.mdc-berlin.de
TargetScan	http://www.targetscan.org/vert_72
**miRNA targets within ORFs**	
TargetScanS	https://tools4mirs.org/software/mirna_databases/targetscans
**miRNAs targets for genomic**	
miRanda	http://cbio.mskcc.org/microrna_data/miRanda-aug2010.tar.gz
miSTAR	http://www.mi-star.org
RNA22	https://cm.jefferson.edu/data-t
miRcode	http://www.mircode.org/index.php
miRDB	http://www.mirdb.org
Bibiserv	https://bibiserv.cebitec.uni-bielefeld.de
**miRNA targets with the secondary structure of the target mRNA**	
PITA	https://genie.weizmann.ac.il/pubs/mir07/mir07_data.html
**miRNA targets based on experimental data**	
starBase	http://starbase.sysu.edu.cn
miRTarBase	http://mirtarbase.mbc.nctu.edu.tw/php/index.php
TarBase	https://carolina.imis.athena-innovation.gr/diana_tools/web/index.php?r=tarbasev8%2Findex
miRanda	http://cbio.mskcc.org/microrna_data/miRanda-aug2010.tar.gz
**Integrated miRNA predicition of mRNA targets**	
Diana	http://diana.imis.athena-innovation.gr/DianaTools/index.php
miRecords	https://tools4mirs.org/software/mirna_databases/mirecords
miRWalk2	http://zmf.umm.uni-heidelberg.de/apps/zmf/mirwalk2/
miRSystem	https://tools4mirs.org/software/target_functional_ analysis/mirsystem
miRdip	http://ophid.utoronto.ca/mirDIP

Several limitations need to be eliminated before the clinical application of miRNA. The phenotypes and molecule responses in mice administered with antagomirs or miRNA mimics were different from or even contrary to those detected from the knockout mice according to the aforementioned description (Lucas et al., 2018). Several possibilities might explain these findings. First, general antagomirs and miRNA mimics in all tissues may not only influence the target of interest in the given cell type but also other potential targets in different cells and tissues. For example, PTEN regulated PI3Ks and AKT to function as an antiapoptotic regulator in diverse cellular types (Oudit, 2004). The downregulation of PTEN mediated by miR-21 in the heart inhibited CM and EC apoptosis, which played a protective role in heart function while promoting VSMC and CF proliferation, resulting in the deterioration of cardiac function. Furthermore, we should also consider the fact that the target of interest may also be regulated by multiple miRNAs before the systemic intervention. PDCD4, one of the targets of miR-21, was demonstrated to be a tumor-suppressor gene and the target of procancer miRNAs (Lankat-Buttgereit and Göke, 2009). Second, anti-miRs may exert non-negligible off-target effects. The enhancement of miRNA levels might affect irrelevant targets. Finally, a short period and partial suppression of miRNA is certainly different from miRNA gene knockout. Gene knockout would cause embryonic phenotypes, which are unlike adult disease etiologies. Moreover, complete gene knockout would cause the organism to initiate a compensation response. To verify the specific cellular effect of miR-21 on cardiac function in CVDs, we must overcome the barriers to effectively and accurately express molecules in a given cell in the cardiovascular system.

## Author Contributions

BD and FW conceived of and designed the review and contributed to the writing of the manuscript. XN, HD, YZ, ZY, HL, JF, and ZW helped with designing the review. DW and CC conceived of and designed the experiments, supervised and contributed to the writing of the manuscript. All authors read and approved the final manuscript.

## Conflict of Interest

The authors declare that the research was conducted in the absence of any commercial or financial relationships that could be construed as a potential conflict of interest.

## Supplementary Material

Supplementary Table 1 The reported expression patterns and functions of miR-21 in CVDs.
