# Supplementary material for: The Cell Type–Specific Functions of miR-21 in Cardiovascular Diseases
Source: Front Genet. 2020 Nov 20;11:563166. doi: 10.3389/fgene.2020.563166 (PMC7714932; doi:10.3389/fgene.2020.563166)
Supplement: Supplementary Table 1 — The reported expression patterns and functions of miR-21 in CVDs. [file Table_1.docx]

**Supplemental Table 1.** The reported expression patterns and functions of miR-21 in CVDs.

| **CVDs** | **Expression** | **Sources** | **Targets** | **Effects** | **Cardiac function** | **References** |
| --- | --- | --- | --- | --- | --- | --- |
| I/R injury | Increase | Heart |  | Mitochondrial NADH⭣, p-AKT⭣, p-eNOS⭣ | Decreased recovery of cardiac function | Anesthesiology. 2015;123:786-98. |
| I/R injury | Decease | Heart |  | pro-apoptotic genes⭡, anti-apoptotic genes⭣ | Increased anti-apoptotic | FEBS Lett. 2008;582:4137-42. |
| I/R injury | Increase | CFs | PTEN | PTEN⭣, MMP-2⭡ | Increase cardiac fibrosis | Cardiovasc Res. 2009;82:21-9. |
| I/R injury | Decease | CMs/CFs | FASL, PTEN | PTEN⭡, FASL⭡, p-AKT⭡ | Decreased infarct size, and ameliorated HF | J Biol Chem. 2010;285:20281-90 |
| I/R injury | Increase | MSCs-exo | PTEN | PTEN⭣, p-AKT⭡ | Increased anti-apoptotic | PLoS One. 2018;13:e0191616 |
| I/R injury | Increase | hMSCs-exo |  |  | Increased cardiac contractility and restored calcium handling | Atherosclerosis. 2019;285:1-9.  Circ Res. 2018;122:933-44. |
| I/R injury | Increase | Heart |  | eNOS⭡, HSP70 ⭡ | Cardio protection | Circ Res. 2009;104: 572-5 |
| MI | Increase | Heart |  |  |  | Proc Natl Acad Sci U S A. 2008;105:13027-32 |
| MI | Increase | CFs | JAGGED1 |  | Increased cardiac fibrosis | J Cell Mol Med. 2018;22:3816-24 |
| MI | Decease | Heart |  | TLR4/NF-κB⭡ | Increased apoptosis and inflammatory | Eur Rev Med Pharmacol Sci. 2018;22:7928-37 |
| MI | Increase | Heart | KBTBD7 | p38/NF-κB signaling⭣ | Attenuated inflammation | Cell Death Dis. 2018;9:769 |
| MI | Increase | CFs | SMAD7 | SMAD7⭣ | Increased cardiac fibrosis | Cell Physiol Biochem. 2017;42:2207-19 |
| MI | Increase | Heart |  |  |  | Physiol Genomics. 2011;43:1087-95 |
| MI | Increase | Exosomal | PTEN | PTEN⭡, p-AKT⭣, BCL-2⭣, VEGF⭣ | Cardio protection | Stem Cells Transl Med. 2017;6:209-22 |
| MI | Increase | Exosomal | PELI1, FASL, PDCD4, PTEN | Anti-cell death activity⭡ | Cardio protection | J Mol Cell Cardiol. 2018;119:125-37 |
| MI | Increase | Atrial tissues | SPRY1 |  | Increased atrial fibrosis | Circ Arrhythm Electrophysiol. 2012;5:1027-35 |
| MI | Increase | CFs | PTEN | PTEN⭣, Akt ⭡, MMP-2⭡ | Increased cardiac fibrosis | Cardiovasc Res. 2009; 82: 21-9 |
| MI | Decease in infarcted areas Increase in border areas | Heart | PDCD4 | AP-1⭣ | Increased anti-apoptotic | J Biol Chem. 2009;284:29514-25. |
| Myocardial remodeling | Depletion | Non-CMs |  |  | Prevented cardiac remodeling and development | Mol Ther. 2016;24:1939-48. |
| HCM | Increase | Plasma |  |  | Correlation with the hypertrophy index | J Am Coll Cardiol. 2014;63:920-7. |
| TAC | Increase | Heart |  |  |  | Circulation. 2010;122:993-1003. |
| Hypertrophy | Increase | Heart |  |  | Protected CMs hypertrophy | J Mol Cell Cardiol. 2007;42:1137-41. |
| Hypertrophy | Increase | Heart | SPRY2 |  | Protected LV hypertrophy | Am J Physiol Endocrinol Metab. 2018;315:E1154-67. |
| Hypertrophy | Increase | Heart |  |  | Protected CMs hypertrophy | Am J Pathol. 2007;170:1831-40. |
| Hypertrophy | Decease | Serum |  |  |  | Kardiol Pol. 2018;76:1009-11 |
| Hypertrophy | Increase | Heart |  |  |  | Proc Natl Acad Sci U S A. 2006;103:18255-60. |
| Hypertrophy | Increase | Heart | SPRY2 | SPRY2⭣ | Protected cardiac hypertrophy | Mol Biol Cell. 2008;19:3272-82. |
| Hypertrophy | Decease/null | Heart |  |  | Failed to response to stress | J Clin Invest. 2010;120:3912-6. |
| Hypertrophy | Increase | CPCs |  |  |  | Cells. 2019;8:1416. |
| Atherosclerosis | Increase | ECs | PPARα | PPARα⭣, VCAM-1⭡,  MCP-1⭡ | Increased EC inflammation | Proc Natl Acad Sci U S A. 2011;108:10355-60. |
| Atherosclerosis | Decease | MPs produced by ECs |  |  | Increased antiapoptotic | Physiol Genomics. 2014;46:833-40. |
| Atherosclerosis | Increase | VSMCs | MMP1 | RECK⭣, MMP-1⭣ | Increased VSMCs dedifferentiation | IUBMB Life. 2018;70:649-57 |
| Arteriosclerosis | Increase | VSMCs | TPM1 | TPM1⭣ | Increased proliferation | Arterioscler Thromb Vasc Biol. 2011;31:2044-53. |
| In-stent restenosis | Increase | VSMCs | PTEN | PTEN⭣ | Increased proliferation | Arterioscler Thromb Vasc Biol. 2015;35:1945-53. |
| Neointimal Lesion Formation | Increase | VSMCs | PTEN | PTEN⭣, BCL-2⭡, AKT⭡ | Increased proliferation and cell survival | Circ Res. 2007;100:1579-88. |
| Acute coronary syndrome | Increase | Plasma |  |  |  | Medicine. 2019;98:e18049. |
| Vein graft failure | Increase | VSMCs, CFs | PTEN |  | Increased proliferation and cell survival | Eur Heart J. 2013;34:1636-43 |
| DC | Increase | CMs | SPRY1 | SPRY1⭣, MAPK⭡ | Increased cardiac fibrosis | Mol Med Rep. 2014;10:161-8. |
| DC |  | CMs | PPARa | PPARa⭡, PGC-1a⭡, NRF2⭡ | Increased cardiac remodeling | Biochim Biophys Acta Mol Basis Dis. 2018;1864:3322-38. |
| DC | Decease | CMs | GSN | GSN⭣, AKT-eNOS-NO⭡ | Decreased ROS and hypertrophy | Cardiovasc Diabetol. 2018;17:123. |
| DC | Decease | CMs |  | Caspase-3/NF-κB⭣ | Decreased CM apoptosis | Anatol J Cardiol. 2018;20:336-46 |
| DC | Increase | CFs | DUSP8 | p38⭡, c- JNK/SAPK⭡ | Increased cardiac fibrosis | Can J Cardiol. 2014;30:1689-99 |
| Atrial fibrosis | Increase | Left atria | SPRY1 | SPRY1⭣, Rac1⭡, CTGF⭡, LOX⭡ | Increased cardiac fibrosis | Basic Res Cardiol. 2012;107:278. |
| Cardiac fibrosis | Increase | EMCs | PDCD4, SPRY1 | Fibrogenic EMT⭡ | Increased cardiac fibrosis | PLoS One. 2013;8:e56280. |
| Cardiac Fibrosis | Increase | ECs | PTEN, PDCD4, TIMP3, RECK | Endothelial markers⭣, EndMT process⭡ | Increased cardiac fibrosis | Arterioscler Thromb Vasc Biol. 2012;32:361-9. |
| Cardiac fibrosis | Increase | Heart | TGF RIII | TGFRIII⭣, TGF-β1, p-SMAD3⭡ | Increased cardiac fibrosis | Int J Biochem Cell Biol. 2012; 44:2152-60. |
| Cardiac fibrosis | Increase | Heart | PTEN SMAD7 | PTEN⭣, SMAD7⭣ | Increased fibroblast proliferation | Eur Heart J. 2015;36:2184-96. |
| Cardiac fibrosis | Increase | CFs | MMP-9 | MMP-9⭣, ARB⭣ | Increased cardiac fibrosis | Mol Cell Endocrinol. 2018;472: 149-58. |
| HF | No change | Heart |  |  |  | Proc Natl Acad Sci U S A. 2006; 103:18255-60. |
| HF | Increase | Heart |  |  | Increased cellular hypertrophy and fetal gene program | Circulation. 2007;116:258-67 |
| Failing RV | Increase | RV CFs | PTEN PDCD4 |  | Reduced RV proliferation | Circ Heart Fail. 2020;13:e006426. |
| Cardiac allografts | Increase | Heart | PTEN | CCL2⭡, PTEN/AP-1⭡ | Increased cardiac fibrosis | Cardiovasc Res. 2016;110:215-26. |
| Failing heart | Increase | CFs | SPRY1 | ERK–MAPK⭡, SPRY1⭣ | Increased cardiac fibrosis | Nature. 2008;456:980-4. |
| Hypertension | Increase | Heart mitochondrial | MT-CYTB | MT-CYTB⭡, MT-ROS⭣ | Reduced blood pressure and cardiac hypertrophy | Circulation. 2016;134:734-51. |
| Shear stress | Increase | ECs | PTEN | PTEN⭣ | Decreased EC apoptosis, increased eNOS and NO production | Biochem Biophys Res Commun. 2010;393:643-8. |

CVD, cardiovascular disease; HF, heart failure; I/R, ischemia/reperfusion; MI, myocardial infarction; AMI, acute myocardial infarction; AF, atrial fibrillation; CMs, cardiomyocytes; ECs, endothelial cells; VSMCs, vascular smooth muscle cells; NF- κB, nuclear factor kappa B; VEGF, vascular endothelial growth factor; CTGF, connective tissue growth factor; LOX, lysyl oxidase; FasL, Fas ligand; HIF-1α, hypoxia-inducible factor 1 alpha; TGF-β, transforming growth factor-β; PDCD4, programmed cell death 4; PTEN, phosphatase and tensin homolog; SPRY1, sprouty homologue 1; ECM, extracellular matrix; MMPs, matrix metalloproteinases; ROS, reactive oxygen species; EndMT, epithelial-to-mesenchymal transition; TPM-1, tropomyosin 1; ADRA2B, adrenal a2B-adrenergic receptor; PPARα, peroxisome proliferators- activated receptor-α; AP-1, activator protein-1; VCAM-1, vascular cell adhesion molecule-1; MCP-1, monocyte chemotactic protein-1; NO, nitric oxide; STAT3, signal transducer and activator of transcription; DUSP8, dual specific phosphatase 8; GSN, gelsolin; BCL2, B cell lymphoma 2; PI3K, phosphoinositol 3-kinase; HDAC8, histone deacetylase-8; TGFβRIII, transforming growth factor beta receptor III; IL-6, interleukin-6; TIMP3, tissue inhibitor of metalloproteinases-3; RECK, Reversion- inducing-cysteine-rich protein with kazal motifs; MT-CYTB, mitochondrially Encoded Cytochrome B; BMP, bone morphogenetic protein; BMPR2, bone Morphogenetic Protein Receptor Type 2; OGD, oxygen–glucose-deprivation; MSC, mesenchymal stem cells; PDGF, platelet-derived growth factor; NADH, nicotinamide adenine dinucleotide (NAD) + hydrogen (H); p-AKT, phosphorylation protein kinase B; p-eNOS, phosphorylation of endothelial nitric-oxide synthase; HSP70, 70-kDa heat shock proteins; TLR4, toll-like receptor 4 precursor; KBTBD7, Kelch Repeat And BTB Domain Containing 7; p38, p38 mitogen-activated protein kinases; SMAD7, SMAD Family Member 7; PELI1, Pellino E3 Ubiquitin Protein Ligase 1; NRF2, nuclear factor erythroid 2-related factor 2; c-JNK/SAPK, c-Jun N-terminal kinase/stress-activated protein kinase; Rac1, ras-related C3 botulinum toxin substrate 1; TGF-beta RIII, transforming growth factor-beta receptor III; TGF-β1, Transforming growth factor beta 1; ARB, angiotensin receptor blockers; CCL2, C-C Motif Chemokine Ligand 2.
